# Bacteria, Protists, and Fungi May Hold Clues of Seamount Impact on Diversity and Connectivity of Deep-Sea Pelagic Communities

**DOI:** 10.3389/fmicb.2022.773487

**Published:** 2022-04-08

**Authors:** Rongjie Zhao, Feng Zhao, Shan Zheng, Xuegang Li, Jianing Wang, Kuidong Xu

**Affiliations:** ^1^Laboratory of Marine Organism Taxonomy and Phylogeny, Shandong Province Key Laboratory of Experimental Marine Biology, Center for Ocean Mega-Science, Institute of Oceanology, Chinese Academy of Sciences, Qingdao, China; ^2^University of Chinese Academy of Sciences, Beijing, China; ^3^Laboratory for Marine Biology and Biotechnology, Pilot National Laboratory for Marine Science and Technology (Qingdao), Qingdao, China; ^4^Institute of Oceanology, Chinese Academy of Sciences, Qingdao, China

**Keywords:** seamount effect, stochastic processes, deterministic processes, pelagic microbes, connectivity

## Abstract

The topography and hydrography around seamounts have a strong influence on plankton biogeography. The intrinsic properties of various biological taxa inherently also shape their distribution. Therefore, it is hypothesized that different pelagic groups respond differently to effects of seamounts regarding their distribution and connectivity patterns. Herein, bacterial, protist, and fungal diversity was investigated across the water column around the Kocebu Guyot in the western Pacific Ocean. A higher connectivity was detected for bacteria than for protists and an extremely low connectivity for fungi, which might be attributed to parasitic and commensal interactions of many fungal taxa. The seamount enhanced the vertical connectivity of bacterial and protist communities, but significantly reduced protist connectivity along horizontal dimension. Such effects provide ecological opportunities for eukaryotic adaption and diversification. All the bacterial, protist, and fungal communities were more strongly affected by deterministic than stochastic processes. Drift appeared to have a more significant role in influencing the fungal community than other groups. Our study indicates the impact of seamounts on the pelagic community distribution and connectivity and highlights the mechanism of horizontally restricted dispersal combined with vertical mixing, which promotes the diversification of eukaryotic life.

## Introduction

Seamounts, which are generally defined as undersea topographic structures with over 1,000 m height, constitute widespread and prominent features of the underwater landscape (Wessel et al., 2007; Staudigel et al., 2010). The specific topography of seamounts creates distinct habitats characterized by their own particular hydrography and mostly hard substrates (Ramirez-Llodra et al., 2010; Rogers, 2018). The interaction of seamount topography and hydrography generates changes in physical oceanographic conditions, such as enhanced vertical mixing, internal waves, Taylor columns, current acceleration, and mesoscale ocean eddies (Chapman and Haidvogel, 1992; Lavelle and Mohn, 2010; Read and Pollard, 2017; Sonnekus et al., 2017; Van Haren et al., 2017). These conditions directly or indirectly enrich the concentrations of particle organic matter and inorganic nutrients and thus promote the metabolic ability of microbes inhabiting seamounts (Genin and Boehlert, 1985; Boehlert, 1988; Rogers, 2018). Seamounts generally harbor higher biomass compared to surrounding waters and have been frequently considered “hotspots” of marine life (Morato et al., 2010). In pelagic systems, immeasurable attention was mainly paid to zooplankton, micronekton and fish ecology (Boehlert, 1988; Genin, 2004; Clark et al., 2010). Bacteria, protists, and fungi are numerically and functionally important microbes in oceans and play fundamental roles in ecosystem functions and biogeochemical processes (Falkowski et al., 2008; Delong, 2009; Massana, 2011). However, the microbial ecology and the underlying ecological processes of seamount ecosystems have received limited attention for research (Vilas et al., 2009; Mendonca et al., 2012).

Bacterial communities in upper waters around seamounts are predominantly controlled by water masses and correlate with sampling depth, while those in deep waters are more homogeneous due to the strong mixing caused by local disruptions of currents by seamounts (Djurhuus et al., 2017). This is supported by the finding that marine currents could improve the dispersal ability of planktonic bacteria (Hanson et al., 2012; Villarino et al., 2018). Considering patterns related to water masses, a previous study showed much higher spatial heterogeneity in pelagic microbial communities around the seamount than previously recognized (Busch et al., 2020). This kind of imprint on pelagic microbial community composition was considered as a “seamount effect” *sensu* lato. Nevertheless, the patterns and underlying mechanisms of microbial dispersal around seamounts remain hardly known.

The microbial communities in natural ecosystems are generally shaped by deterministic and stochastic processes (Chase, 2010; Vellend, 2010; Zhou and Ning, 2017). Deterministic processes, comprising variable selection and homogeneous selection, result from the abiotic environmental factors and biotic interactions between individuals. Stochastic processes due to random changes (birth, death, immigration and emigration, spatiotemporal variation, and/or historical contingency) in community structure consist of dispersal limitation, homogeneous dispersal, and drift (Stegen et al., 2013). For example, the Tara Oceans project revealed that picoeukaryotes in the global surface water are predominantly structured by dispersal limitation, while bacteria appeared to be shaped by the combined action of dispersal limitation, drift, and selection (Logares et al., 2020). Furthermore, the “size-plasticity” hypothesis was advocated by a different study highlighting the conclusion that protist communities in the East China Sea were more strongly governed by species sorting relative to dispersal limitation across different water depths than their bacterial counterparts (Wu et al., 2018). In the seamount areas, apart from physical relationships between water masses and seamounts, the drivers of pelagic microbial distribution are closely associated with the intrinsic properties of microorganisms.

Bacteria, protists, and fungi exhibit differences in body size, abundance, and dispersal potential, and these traits may shape their patterns in distribution. We hypothesize that pelagic bacteria, protists, and fungi respond differently to the seamount effect regarding their diversity, distribution, and connectivity patterns and also the underlying drivers. The diversities of bacteria, protists, and fungi were explored along the water column above and around the Kocebu Guyot, a flat-topped seamount in an oligotrophic tropical area of the western Pacific Ocean. Connectivity refers to the exchange of individuals across separated subpopulations that comprise a metapopulation (Cowen et al., 2007; Villarino et al., 2018). In this article, we take the seamount pelagic community as a whole metapopulation and pelagic microbes from different water layers or sites as subpopulations. To explore connectivity between samples from each water layer, the network analysis, which has been used in the benthic fauna, the planktonic larvae and fossil record (Kiel, 2016, 2017; Ristova et al., 2017; Smith et al., 2017; Mullineaux et al., 2018), was performed. Our study aimed to (i) determine the distribution and connectivity patterns of bacteria, protists, and fungi around the seamount and (ii) explore the processes driving the observed distribution patterns.

## Materials and Methods

### Sample Collection

**Figure 1:**
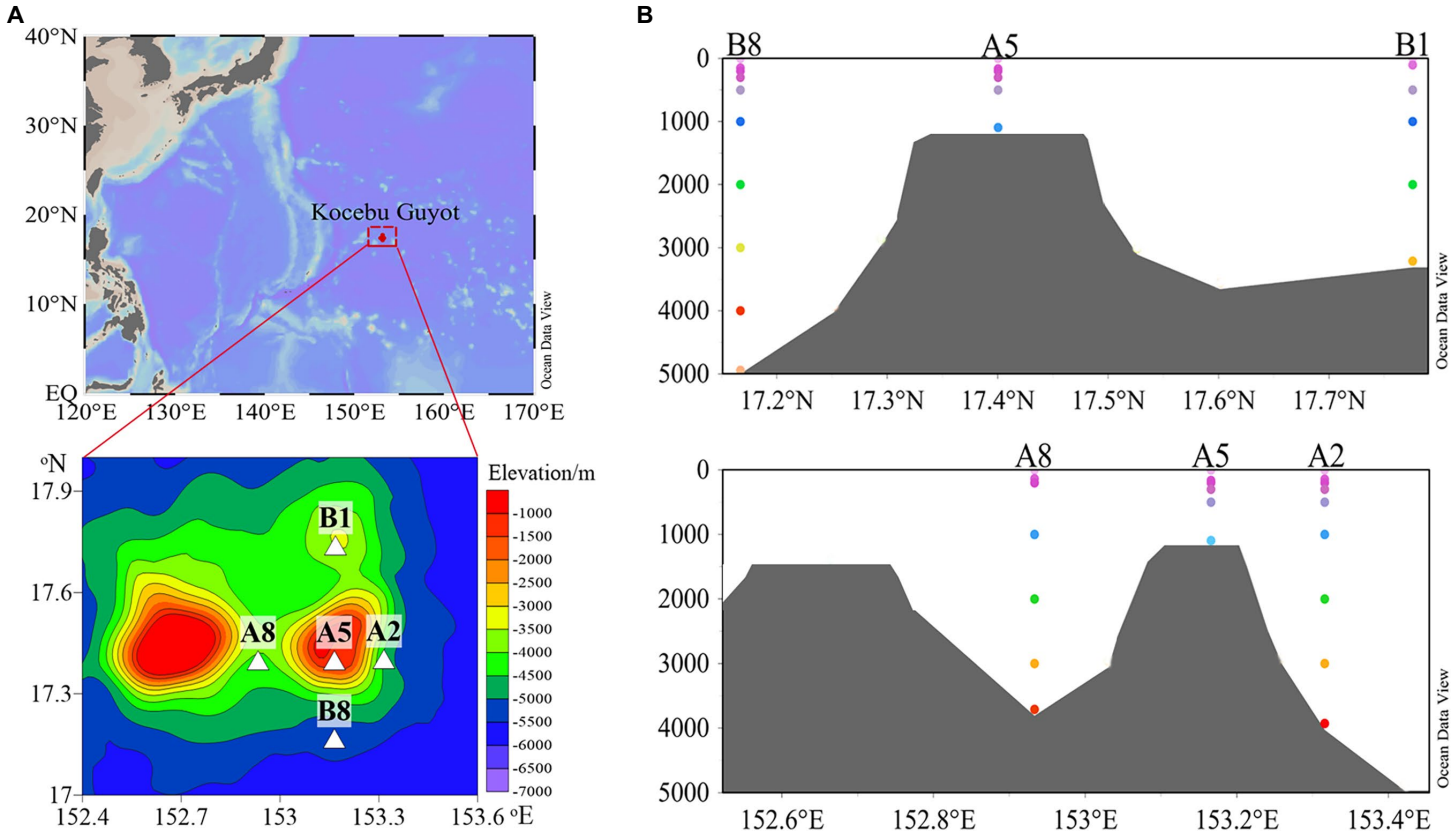
**(A)** Contour map showing the location of sampling sites and **(B)** sectional views of the Kocebu Guyot, where colored dots indicate water depth for each sampling site.

Water samples were collected at the Kocebu Guyot in the Magellan Seamount chain located in the oligotrophic tropical western Pacific Ocean. The Kocebu Guyot is an approximately 4,300-m-high flat-topped deep-sea mountain with the shallowest summit at 1,150 m depth. During the cruise of R/V *KeXue* in 2018, two sections which crossed at the top of seamount were established to carry out investigation (Figure 1). Station A5 was located above the seamount, and the other four sites were crossly situated around the seamount along or perpendicular to water currents. Particularly, station A8 was located between two flat-topped seamounts with a similar peak depth. The greatest depths at A2, A8, B1, and B8 were 3,900, 3,500, 3,200, and 5,003 m, respectively. Thirty-eight seawater samples were collected from five sampling stations along the vertical profile, and samples were named based on sampling depth (Supplementary Table S1).

A total of 20 L seawater was collected from each water layer at all sampling stations with Niskin Bottles attached to a rosette equipped with Seabird CTD probes. Samples were pre-filtered first through a 200-μm sieve and subsequently filtered through a 0.22-μm polycarbonate filter. Filters were then flash-frozen in liquid nitrogen and stored at −80°C until further processing.

Water temperature (T) and salinity (S) were measured directly using probes *in situ*. Dissolved oxygen (DO) was also measured *in situ* by Winkler iodometry at ≤2% relative standard deviation after fixation with manganese sulfate and alkaline potassium iodide solution (Ma et al., 2020a). Chlorophyll *a* was extracted in 90% acetone at 4°C for 24 h, and quantified using a Turner Designs Trilogy Fluorometer (Turner Designs, United States; Parsons, 1984). Nutrient concentrations in the GF/F filtrate were determined with a continuous flow analyzer (QuAAtro, Seal Analytical Limited, United Kingdom) according to the Joint Global Ocean Flux Study (JGOFS) spectrophotometric method (Gordon et al., 1993). Particulate organic carbon (POC) concentrations collected by GF/F filters were measured by using elemental analyzer combined with stable isotope ratio mass spectrometer (EA-IRMS, Thermo Fisher Scientific Flash EA 1112 HT- Delta V Advantages, United States).

### DNA Extraction and PCR Amplification

Environmental DNA was extracted from seawater samples using the All Prep DNA/RNA Mini Kit (Qiagen, Germany) following the manufacturer’s instructions.

Specific primers and PCR protocols were utilized to amplify different target sequences. In order to amplify 16S rDNA sequences in bacteria, forward primer U341F (Hansen et al., 1998) and reverse primer R685 (Wang and Qian, 2009) were set. The hypervariable V4 region of protist 18S rDNA was amplified using a set of eukaryote-specific primers Reuk454FWD1 and TAReukREV3 (Stoeck et al., 2010). For the detection of fungi, the ITS2 (Internal transcribed spacer 2) region was amplified using specific primers ITS3 and ITS4 (White et al., 1990). Each sample was analyzed in three PCR replicates for each primer pair to minimize PCR bias before prepping sequence libraries. A total of 35 and 38 samples were amplified successfully by the 16S and 18S primers from five sample sites, respectively (Supplementary Table S1). Only 26 samples from three sites (A2, A8, and B8) were amplified successfully with the ITS region primers (Supplementary Table S1).

### High-Throughput Sequencing and Data Processing

The target regions of 18S/16S rRNA gene and ITS2 were sequenced by the Illumina MiSeq platform (Illumina, Inc., San Diego, CA, United States) using the previously described 2 × 250 bp paired-end protocol. Further library preparation was carried out with the addition of standard Nextera indexes (Illumina, Inc., San Diego, CA, United States) using NEB Next Ultra DNA Library Prep Kit for Illumina (NEB, United States). These sequence data have been submitted to the GenBank databases under accession number PRJNA700132. To compare protists in seamount and non-seamount areas, the non-seamount dataset of protist 18S rDNA sequence retrieved from Zhao et al. (2020) was also included and analyzed.

The obtained sequences were demultiplexed according to the indexes and internal barcodes applied. The UPARSE pipeline was used for subsequent elaborations, such as excluding duplicates, low-quality and short sequences (<100 bp), singletons (reads occur only once), and possible contaminations (Edgar, 2013). Operational taxonomic units (OTUs) were delineated in UPARSE by a sequence similarity of 97% for all microorganism taxa (Zhao et al., 2019). Taxonomic annotation for protists, bacteria, and fungi was implemented using BLAST with the SILVA (Quast et al., 2013; Yilmaz et al., 2014), RDP (Cole et al., 2005), and UNITE (Nilsson et al., 2019) databases, respectively. Moreover, in the 18S dataset, fungal sequences were too rare to fully demonstrate the fungi community, and thus, fungal taxa in the 18S dataset were excluded. In the 16S dataset, Archaea sequences were too rare to continue and thus discarded. Three independent datasets of protists, bacteria, and fungi were finally put forward for subsequent analysis. Classifications of different microorganism taxa were manually modified (Parte, 2018; Adl et al., 2019).

To address the seamount effect on microorganism communities, we also applied dataset of protists in non-seamount area of the western Pacific Ocean from a previously published article (Zhao et al., 2020).

### Assessment of Alpha and Beta Diversity

The degree of sample saturation was tested through rarefaction curves. The number of sequences per sample was normalized to the smallest sample size using USEARCH (v.10.0.240) to ensure inter-sample comparability and used to calculate alpha diversity (the minimum sample size of bacteria, protists, and fungi was 5,670, 8,937, and 7,438, respectively). The OTU richness of each sample was assessed to interpret the alpha diversity of different water layers. Following a Shapiro–Wilk normality test and Bartlett test of homogeneity of variances, one-way ANOVA was employed to assess the differences in alpha diversity between depth groups, and the Fisher’s least significant difference (LSD) test was sequentially applied for significantly differentiated microbial groups. Data with non-normal distribution were analyzed by the Kruskal–Wallis rank sum test.

Before beta diversity analysis, the three separate OTU tables of bacteria, protists, and fungi were first adjusted with zComposition package and implemented with centered log-ratio (CLR) transformation with CoDaseq package in R (v.4.1.0; Martin-Fernandez et al., 2015; Palarea-Albaladejo and Antoni Martin-Fernandez, 2015; Gloor and Reid, 2016; Quinn et al., 2019). The Aitchison distances of samples were calculated using the CLR-transformed OTU table with CoDaseq package (Gloor et al., 2017), then a basic hierarchical agglomerative clustering of samples based on the Aitchison distance matrix was implemented, and samples were partitioned into several depth-based groups.

### Evaluation of Community Connectivity by Network Analysis

To explore interactions between samples from each water layer, a network analysis considering samples as objects was conducted based on the CLR-transformed OTU data for each sample.

The *Φ* correlation of samples (Lovell et al., 2015; Gloor et al., 2017) was calculated using CoDaseq package in R (v.4.1.0) and visualized using Gephi (v.0.9.2; Mathieu et al., 2009). With the aim to reflect robust core interactions from samples, correlations with *Φ* < 0.15 were selected. Network was constructed using (1-phi) as edge weight to reflect the relative level of correlation. In order to eliminate the influence of sample number, additional network analysis of bacteria and protists with the same samples arrangement as fungi was implemented.

### Quantification of the Selection, Dispersal, and Drift

The null model framework was utilized to assess the relative importance of selection, dispersal, and drift on shaping microbial communities by analyzing phylogenetic and taxonomic compositions of these communities. First, in order to infer the action of selection, the representative OTU sequences of protists, bacteria and fungi were applied to construct phylogenetic trees using FastTree (V2.1.11; Price et al., 2010). Afterward, the β-Nearest Taxon Index (βNTI), which refers to the variance between observed βMNTD (abundance weighted β-mean nearest taxon distance) and mean value of null models (999 randomizations), was calculated following Stegen’s methods (Stegen et al., 2013, 2015). βMNTD values higher than expected (βNTI>2) indicate the community is driven by heterogeneous selection, while βMNTD values lower than expected (βNTI<2) indicate the community is driven by homogeneous selection. Second, CLR-transformed OTU data were used to determine the action of dispersal and drift. Chase’s code was utilized to determine the Raup–Crick matrix (β_RC_) based on the OTU occurrence data (Chase et al., 2011). β_RC_ referred to the difference between measured observed number of shared species between two samples and the shared species number that generated from null models (9,999 randomizations). β_RC_ values >0.95 or <−0.95 indicate that community is driven by dispersal limitation or homogeneous dispersal, respectively. In contrast, |β_RC_| < 0.95 indicates that the community is driven by drift. Statistical analyses were implemented using the Picante (Kembel et al., 2010) and Vegan (Dixon, 2003) packages in R (v.3.6.2). We also disentangled the effects of selection, dispersal, and drift on bacterial and protists communities with the same sample arrangement as fungi to exclude the sampling influence.

### Estimation of Effects of Environmental Factors

In order to interpret the environmental factors driving the partitioning of bacterial/protist/fungal diversities, the CLR-transformed OTU abundance table of samples combined with a matrix of log-transformed values of environmental factors (depth, temperature, salinity, concentrations of NO_3_-N, NO_2_-N, NH_4_-N, SiO_3_-Si, PO_4_-P, dissolved oxygen, and chlorophyll *a*) was used for distance-based redundancy analysis (dbRDA) using the vegan package in R (v.3.6.2). Samples lacking data on environmental factors were excluded from this stage of analysis. The significance of RDA module and each axis was determined by a permutation test. Additional RDA analysis was fulfilled to explore the environmental factors influencing bacterial and protists communities with same sample number as fungi.

## Results

### Alpha Diversities and Taxonomic Identification of Different Pelagic Groups

**Figure 2:**
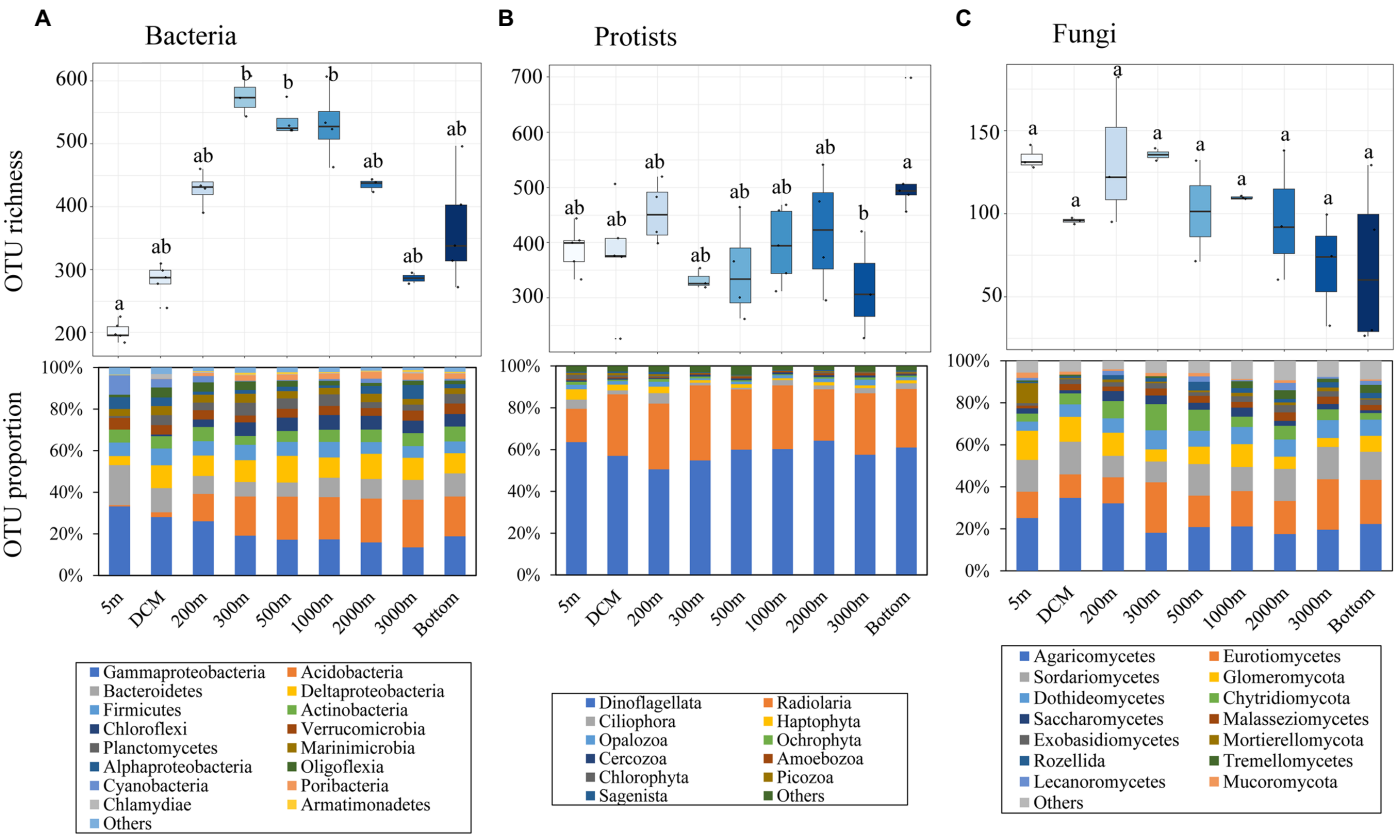
Variations in Operational taxonomic unit (OTU) richness and their corresponding taxonomic identifications (mainly at phylum and class level) along the sampling depth range for **(A)** bacteria, **(B)** protists, and **(C)** fungi. Bottom refers to the max depth of each site. The significant inter-group differences among water layers were annotated above each box with “a,” “b,” or “ab” based on the Fisher’s least significant difference (LSD) test. The phylum/class with the relative OTU proportion more than 0.5% of bacterial, protist, and fungal communities is shown in barplots, and others included taxa with the relative proportion less than 0.5%.

The rarefaction curves of protist samples were seldom saturated, while nearly all of those of bacterial and fungal samples were saturated (Supplementary Figure S1). Following normalization, a total of 2,345, 1,731, and 462 OTUs were detected for bacterial, protist, and fungal communities, respectively. Different pelagic groups presented various patterns of OTU richness along the depth gradient (Figures 2A–C). In brief, bacterial OTU richness presented a unimodal pattern along water depth with its peak value appearing at 300 m and its minimum at the surface, while that of fungi showed a generally decreasing trend. Protist richness exhibited a trimodal pattern with peak values appearing at the 200 m, 2,000 m and bottom layers, and the lowest value at 3,000 m. The OTU richness of protists and bacteria differed significantly (*p* < 0.05) between depth layers, while that of fungi did not show significant variations.

The taxonomic analysis of bacteria, protists, and fungi was performed mainly at the class and phylum levels. The patterns of relative abundance of taxonomic groups varied among water layers (Figure 2). Highly abundant Gammaproteobacteria largely dominated the bacterial community (Supplementary Figure S2A), with a maximum sequence proportion of 74.2% in the bottom water and a minimum of 51.6% in the deep chlorophyll maximum (DCM) water. Different taxa in the protist community showed relatively constant OTU proportions (Figure 2B). With increasing depth, the frequency of Dinoflagellata and Radiolaria OTUs decreased, while those of Opalozoa and Ochrophyta showed the opposite trend. With regard to the fungal community (Figure 2C), the proportion of OTUs of different fungal taxa showed stable distribution patterns. Conversely, the relative abundance of these taxa showed a disordered configuration (Supplementary Figure S2C).

### Cluster Analyses of Detected Pelagic Groups

**Figure 3:**
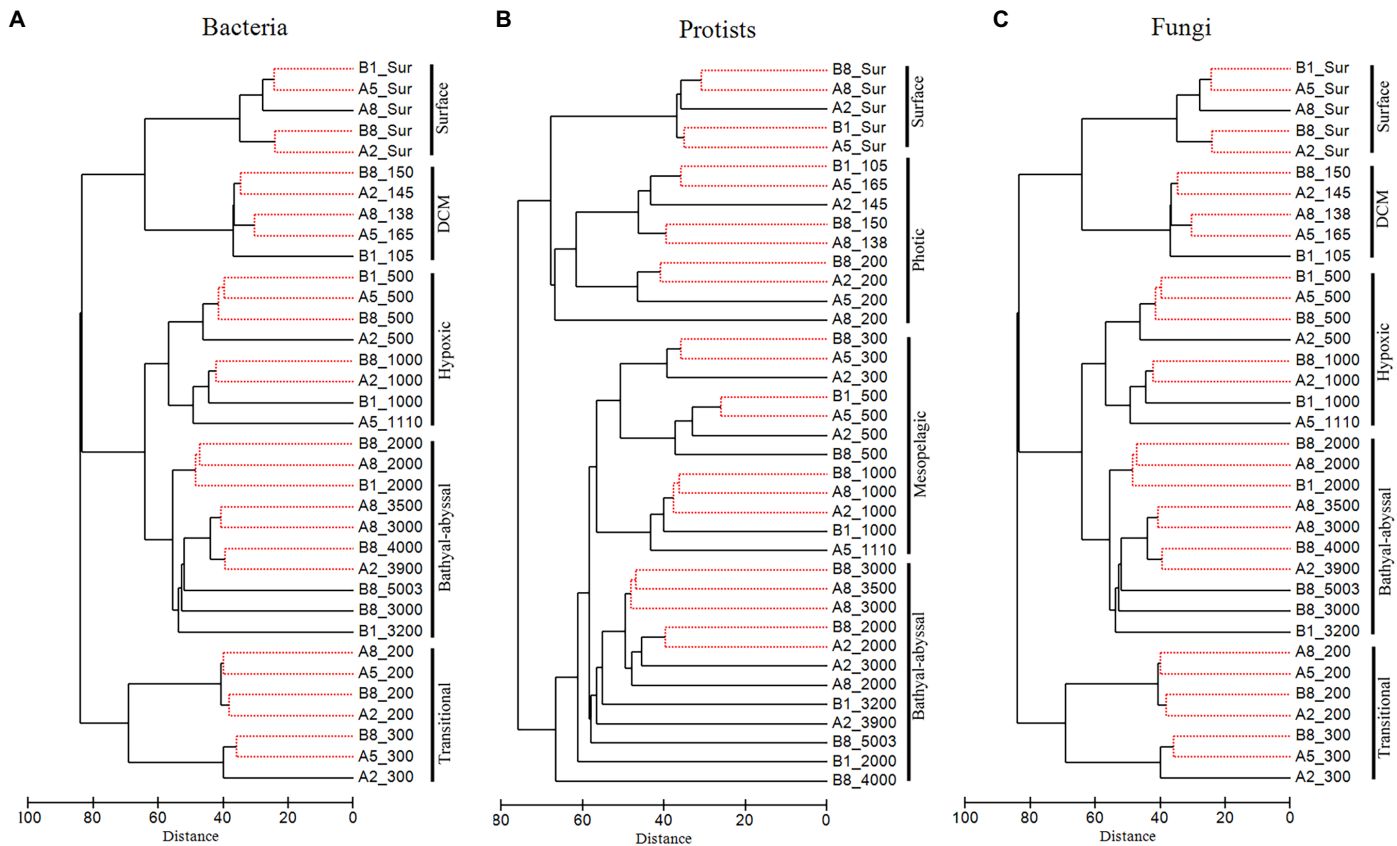
Clustering results for bacteria **(A)**, protists **(B)**, and fungi **(C)** based on the Aitchison distances of samples. No significant differences (SIMPROF, *p* > 0.05) regarding OTU composition and abundance of sequences were detected among samples connected with a red dashed line.

Significant vertical patterns in bacteria, protist and fungal communities were detected based on the Aitchison distances of samples (Figure 3). Samples from the same water layer were generally grouped into one cluster and exhibited high similarity with clusters from adjacent depth layers. Bacterial and fungal samples could be commonly divided into five groups, i.e., surface community, DCM community, transitional community (200–300 m), hypoxic community (500–1,000 m), and bathyal-abyssal community (2,000 m-bottom). Protist communities were grouped somewhat differently, with surface, DCM and 200 m samples clustering as one photic zone community, and samples from 300 m and deeper clustering together.

### Connectivity Among Samples for Different Pelagic Groups

**Figure 4:**
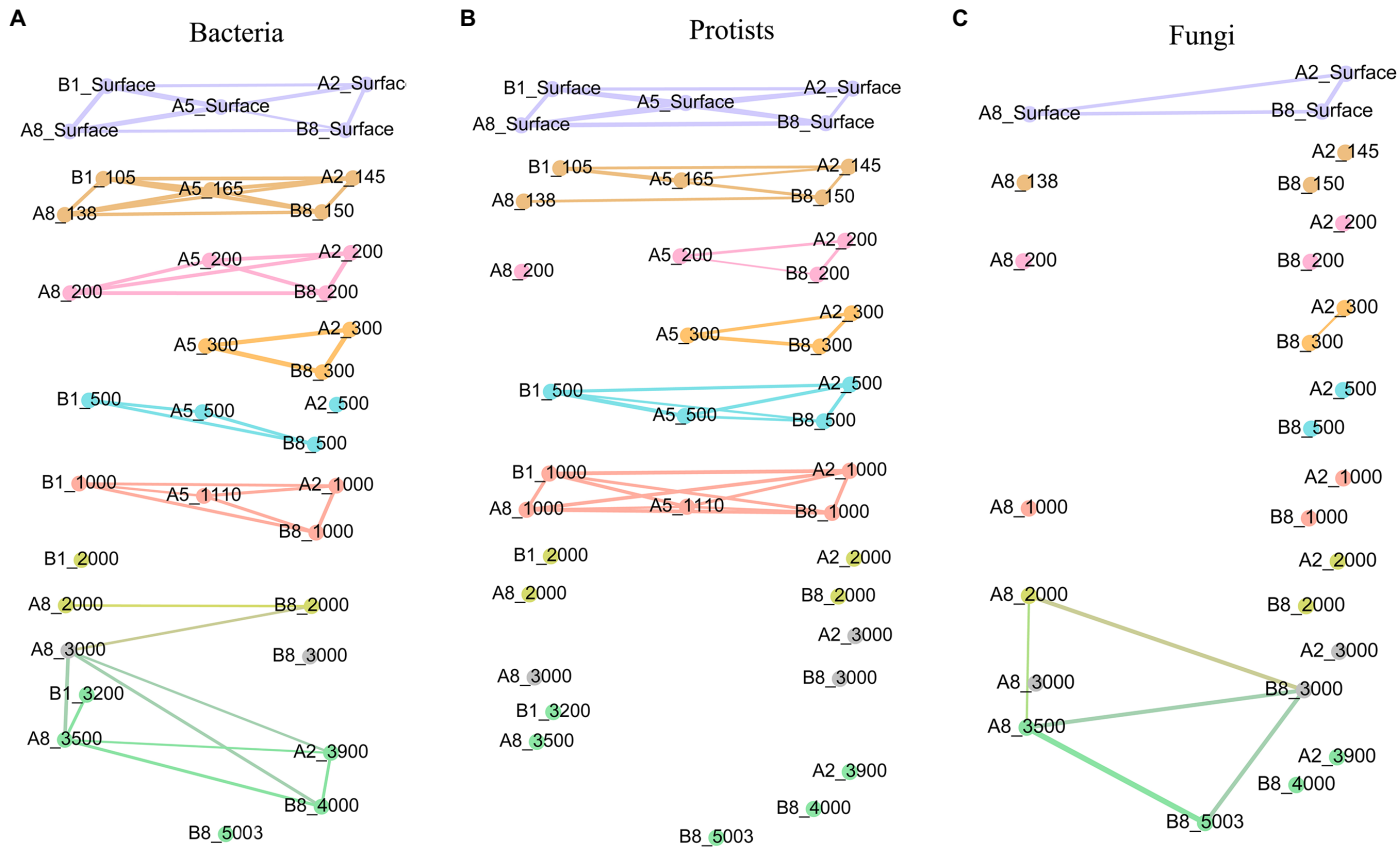
Network of correlations between samples with regard to different microorganism groups: **(A)** bacteria, **(B)** protists, **(C)** fungi. Samples from the same water depth were assigned the same color. Significant correlations (Φ < 0.15) are shown by the lines between samples.

The co-occurrence analysis of communities based on samples from different water layers attributed more intimate intra-layer interactions within bacteria and protists than the fungal community, which was not even connected with the adjacent community (Figure 4).

Among the examined microbial groups, the bacterial network contained the most intimate structure. Samples from the surface, DCM, 200-m and 300-m layers showed group correlations within each layer, whereas samples from layers of 500 m to bottom water connected with others not only from the same water layer but also with those from adjacent vertical layers. The protist network contained several modules comprising samples from within the surface, DCM and 1,000 m water layers above the seamount summit. By contrast, samples below 1,000 m depth did not have strong and significant correlations with those from either the same water layer or other water layers. When compared with those of bacteria and protists, the fungal network possessed fewer correlations, and their group correlations only occurred in surface water layers, 300 m at the A2 and A8 stations, 2,000 m, 3,000 m and bottom water layers at the A8 and B8 stations. A network reflecting the interactions of the whole microbial community in the seamount based on the total OTU information of bacteria, protists, and fungi was further implemented. The network exhibited intra-layer connectivity above the summit depth (1,110 m), while no connection was found under 1,100 m (Supplementary Figure S3).

The modularity indexes established for bacteria, protists, and fungi were 0.832, 0.798, and 0.564, respectively, suggesting that all three networks had modular structures (Newman, 2006). The mean clustering coefficients (the degree of samples tending to cluster together) for bacteria, protists, and fungi were 0.931, 0.944, and 0.905, respectively.

### Quantification of Selection, Dispersal, and Drift Structuring Pelagic Communities

The impact of selection, dispersal, and drift on structuring microbial communities of the Kocebu Guyot was calculated using Stegen’s null model (Supplementary Figure S4). The bacterial community was primarily shaped by variable selection (63.7%), then by homogeneous dispersal (16.7%), dispersal limitation (11.8%) and homogeneous selection (3.7%), thus was mainly affected by deterministic processes. Drift only had 4.2% contribution toward structuring the bacterial community.

Likewise, the protist community was more strongly affected by deterministic than stochastic processes. Specifically, variable selection played a predominant role by accounting for 72.8% in the structuring of protist community followed by homogeneous dispersal (20.1%). The importance of drift (3.1%) and dispersal limitation (2.3%) was similar in structuring the protist community, and homogeneous selection only had 1.7% contribution.

Stochastic processes had higher percentage in shaping the fungal community compared to bacterial and protist communities; nevertheless, the most important ecological process in shaping the fungal community was variable selection with a proportion of 57.5%. Homogeneous dispersal also played a significant role with a proportion of 23.7% and then followed by drift at 15.4%. Meanwhile, dispersal limitation and homogeneous selection accounted for 2.8 and 0.6%, respectively, of the total contribution.

### Selective Forces Acting on Pelagic Communities

The forward selection of environmental factors indicated that NO_3_-N, depth, temperature, chlorophyll *a*, dissolved oxygen, and salinity had considerable driving effects on the protist community (*p* < 0.01), and the bacterial community was also significantly (*p* < 0.01) affected by the above factors with the additional PO_4_-P. The fungal community was considerably influenced by depth, PO_4_-P, chlorophyll *a,* and salinity (*p* < 0.01; Table 1).

**Table 1:** Environmental factors shaping bacterial, protist, and fungal communities, as revealed by RDA analysis.

	Bacteria	Protists	Fungi
Significantly affected factors	NO_3_-N	NO_3_-N	Depth
	PO_4_-P	Depth	PO_4_-P
	Depth	Temperature	Chl *a*
	Temperature	Dissolved oxygen	Salinity
	Chl *a*	Chl *a*	
	Dissolved oxygen	Salinity	
	Salinity		
*R*^2^ (adjusted)	0.616^**^	0.503^**^	0.314^**^

** *p* < 0.01 (significance level of adjusted *R*^2^).

### Dispersal Influencing the Pelagic Communities

The proportions of OTUs shared among samples within each water layer were generally much lower below the seamount summit (1,110 m) than those above it. As for protists in non-seamount areas, the proportions of shared OTUs within the same water layers were much higher than those in seamount for samples either at and above 1,000 m or at and below 2,000 m. In terms of taxonomic groups, the highest ratio of shared OTUs was detected in the bacterial community, while the fungal community had the lowest proportion (Table 2).

**Table 2:** Proportions (%) of OTUs shared among samples within each water layer (horizontal).

		Water layers	Bacteria	Protists	Fungi	Data source
Samples at and above 1,000 m	Seamount	Surface	39.4	34.4	28.3	Present study
		DCM	30.8	19.1	10.4	
		200 m	42.4	25.5	23.3	
		300 m	62.0	42.8	49.6	
		500 m	32.2	29.0	34.2	
		1,000 m	37.8	30.6	16.3	
		Mean	**40.7**	**30.2**	**27.0**	
	Non-seamount	Surface	**-**	51.1	**-**	Zhao et al., 2020
		DCM	**-**	42.9	**-**	
		200 m	**-**	43.2	**-**	
		1,000 m	**-**	73.7	**-**	
		Mean	**-**	**52.8**	**-**	
Samples at and below 2,000 m	Seamount	2,000 m	36.6	21.4	15.2	Present study
		3,000 m	28.8	20.7	20.5	
		Bottom	26.6	21.1	8.3	
		Mean	**30.7**	**21.1**	**14.7**	
	Non-seamount	2,000 m	**-**	25.6	**-**	Zhao et al., 2020
		Bottom	**-**	20.8	**-**	
		Mean	**-**	**23.2**	**-**	

-, data not available. Bold values indicate the mean value of shared proportions.

Along the vertical dimension, the proportion of shared OTUs in samples above the summit from the same site was lower than those below it. Compared with protists in open ocean, the proportion of shared OTUs in seamount was generally higher. Besides, the proportion of shared OTUs in samples from the same site was the lowest for the bacterial community and the highest for the fungal community above the summit, but got reversed below it (Table 3).

**Table 3:** Proportions (%) of OTUs shared between samples from the vertical profile of each site.

		Sites	Bacteria	Protists	Fungi	Data source
Samples at and above 1,000 m	Seamount	A2	3.8	8.3	10.4	Present study
		A5	4.0	11.7	-	
		A8	-	7.4	10.6	
		B1	5.0	7.9	-	
		B8	5.8	11.1	7.9	
	Non-seamount	DY7	-	**4.2**	-	Zhao et al., 2020
		DY12	-	**3.9**	-	
Samples at and below 2,000 m	Seamount	A2	-	31.9	32.5	Present study
		A8	46.3	35.8	37.9	
		B1	36.9	40.8	-	
		B8	31.1	37.1	18.0	
	Non-seamount	DY7	-	**22.6**	-	Zhao et al., 2020
		DY12	-	**21.7**	-	

-, data not available. Bold values indicate the shared proportions of protists in the non-seamount area.

## Discussion

**Figure 5:**
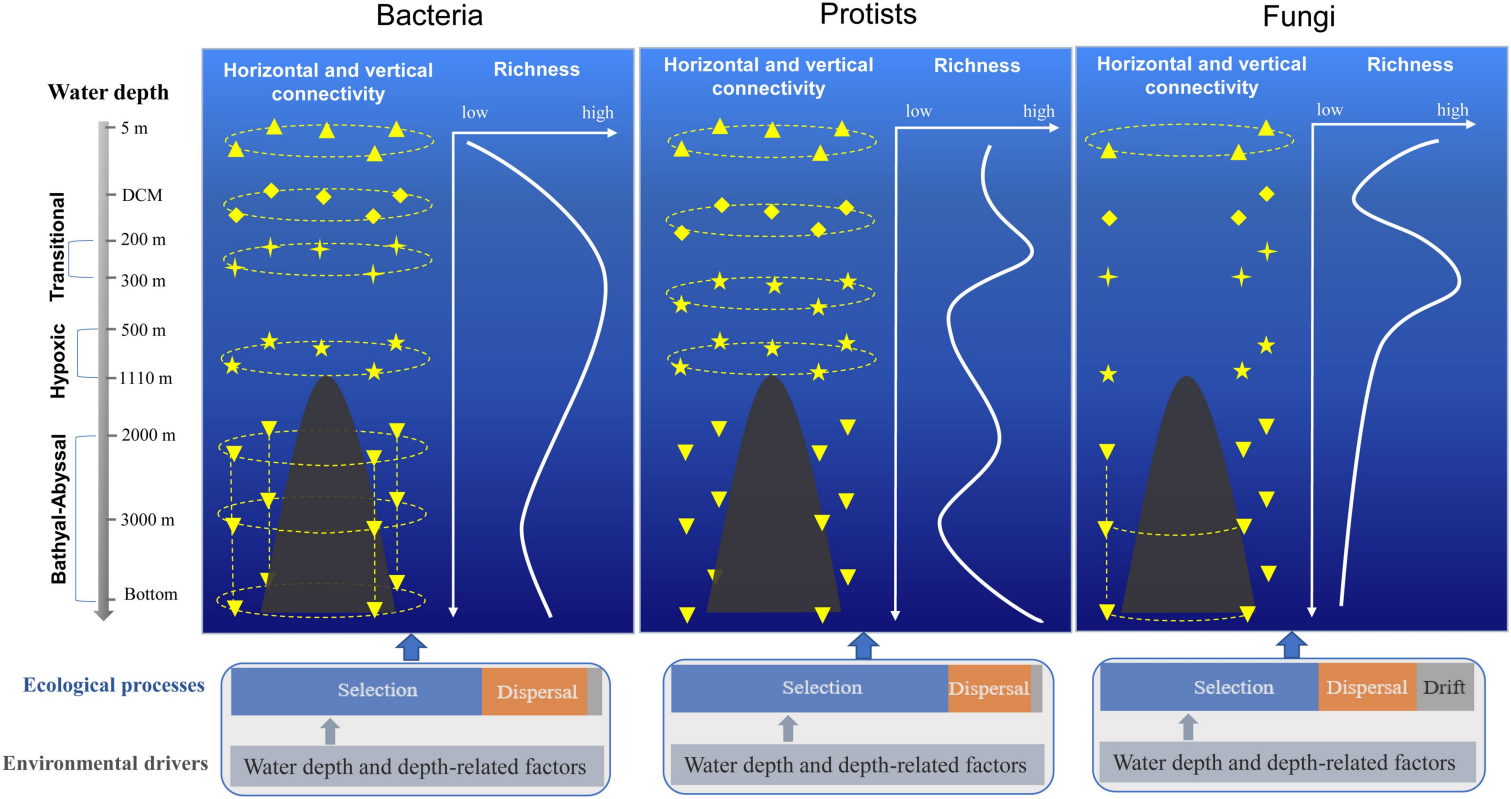
Conceptual model of the seamount effect on diversity, distribution, and connectivity patterns of bacterial, protist, and fungal communities.

Based on the diversity, distribution, and connectivity patterns of bacterial, protist, and fungal communities, a conceptual model of the seamount effect was constructed in this study (Figure 5). Overall, bacterial community exhibited higher level of connectivity between different water layers compared with protists and fungal communities. The horizontal connectivity of protist community below summit depth (1,110 m) decreased due to the hindrance of the seamount. Drift played a more important role in shaping fungal community compared with bacteria and protists.

### Seamount Effect Along the Horizontal Scale

Seamounts in the open ocean theoretically interrupt water flow resulting in physical changes, which are in turn expected to cause variations in microbial distribution. A much higher spatial heterogeneity of bacterial communities was revealed along the water layer slightly above the summit (−600 m) of a seamount by Busch et al. (2020). This kind of imprint on the pelagic microbial community composition was subsequently called “seamount effect” (*sensu* lato). This is a reasonable presumption since the interaction of topography and hydrography around seamounts at shallow and shallow-intermediate depth generates physical oceanographic changes, such as Taylor columns, current acceleration, and mesoscale ocean eddies (Genin, 2004; Lavelle and Mohn, 2010; Read and Pollard, 2017).

However, the occurrence of such “seamount effect” in deep (summit deeper than 1,000 m) seamounts has never been reported. In the present work, the different responses of bacterial, protist, and fungal communities to seamount effect were determined along the horizontal and vertical gradients. The Kocebu Guyot clearly reduced horizontal interactions within the protist community below the seamount summit, where the community connectivity was much lower across the same water layers, compared with that above the seamount summit. This might be attributed to enclosed circulation cells (“sheath-water”) in the deep-water flow created by the seamount (White et al., 2007). This “sheath-water” is retained by the seamount, thereby reducing community connectivity between that and the surrounding deep water (Giljan et al., 2020). Such seamount effect should contribute to eukaryotic diversification, resulting in greater diversity around seamounts than in surrounding deep seas.

By contrast, the bacterial community had a significant number of connections within each intra-layer group throughout the water column. The difference between bacteria and protist distribution might be attributed to variation in their body sizes; bacteria are much smaller than protists and are less constrained by dispersal (Logares et al., 2020).

When compared with bacterial and protist communities, the fungal community exhibited many fewer connections. Notably, the number of samples involved in the fungal community analysis was lower than that for bacteria and protists. Subsequently, extra analyses for protists and bacteria were undertaken with the same sample arrangement as for fungi, and results were consistent with results of original analyses (Supplementary Tables S1, S2; Supplementary Figures S5, S6). This low connectivity of the fungal community might be attributed to the parasitic and commensal trophic mode of many members, leading to different responses to the seamount effect (Amend et al., 2019).

The detected variations in responses of different taxonomic groups to this effect might be also attributed to the intrinsic properties of each taxon. These data indicate complex distribution patterns within different pelagic communities around the seamount, hence further studies are necessary to individually analyze taxa with different body size and trophic type for an improved understanding of their respective distribution and connectivity patterns.

### Seamount Effect Along the Vertical Gradient

Topography and hydrography interact around seamounts to generate enhanced vertical mixing (Lavelle and Mohn, 2010; Read and Pollard, 2017); thus, it structures pelagic richness, community composition, and connectivity. Surprisingly, no change induced by the seamount was detected by this study in the general patterns of bacterial and fungal species richness. Bacterial OTUs exhibited the highest richness in the layer between the oxygen rich zone and the oxygen minimum zone (OMZ), which is consistent with previous results of Walsh et al. (2016) and Moeseneder et al. (2001). Likewise, the same variation trend in fungal richness was detected in the western Pacific Ocean from epi- to abyssopelagic zones (Li et al., 2019) and around the seamount (this study), which generally decreased with water depth.

**Figure 6:**
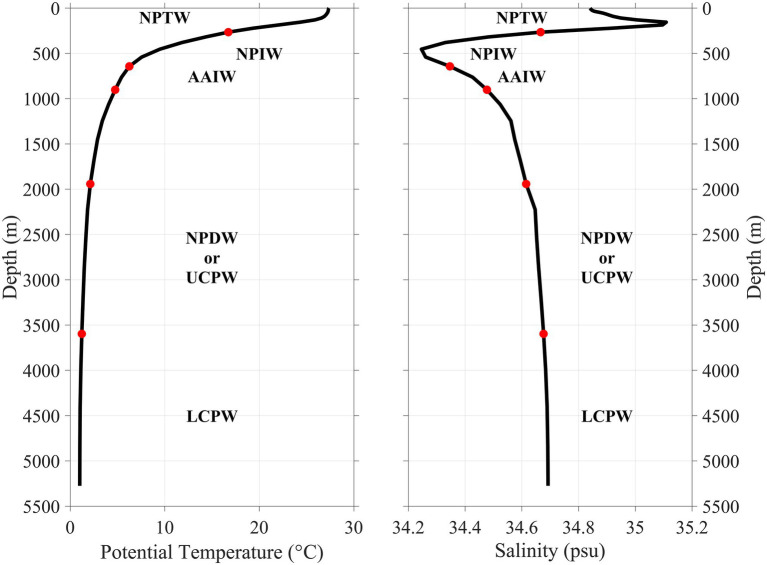
Depth distribution of Antarctic Intermediate Water (AAIW), North Pacific Deep Water (NPDW), and Lower Circumpolar Water (LCPW) and their corresponding potential temperature and salinity in the surrounding area of the Kocebu Guyot. Mean temperature and salinity profiles over the measurement period are from a data assimilative global ocean analysis product (PSY4V3R1) created by Mercator Ocean. Other abbreviations: NPTW, North Pacific Tropical Water; NPIW, North Pacific Intermediate Water; and UCPW, Upper Circumpolar Water.

Regarding protists, we revealed an unusual trimodal pattern of OTU diversity around the seamount, with the highest diversity in the bottom (3,500–5,000 m) water layer, the second highest in the 200-m layer and the third highest in the 2000-m layer. This finding contrasts with the result of Zhao et al. (2017), who observed a generally unimodal diversity pattern of protist ciliates along the water column of about 5,000 m in the western Pacific Ocean, with the highest OTU diversity in the DCM layer and the second highest in the 200 m layer. Food limitation is often one of the most significant factors regulating the abundance and diversity of deep-sea eukaryotes. Usually, the diversity of eukaryotes is relatively low in deep water layers due to the presence of less labile organic matter (Schnetzer et al., 2011). Therefore, the peak diversity values in the bottom (3,500–5,000 m) and 2000-m water layers are unusual. This vertical pattern is consistent with the local mixture of the Antarctic Intermediate Water (AAIW) and North Pacific Deep Water (NPDW) at about 2,000 m and the advection of Lower Circumpolar Water (LCPW) originated from the Antarctic at about 5,000 m (Figure 6; Wang et al., 2021). A unimodal (Thresher et al., 2014) or bimodal (Henry et al., 2014) diversity pattern of megabenthos on deep seamounts was also observed and was attributed to the input of different water masses which fostered the abundance and species richness of megabenthos.

With respect to community composition, it is reasonable that advection by water flow carries extra organic matter facilitating bacterial growth in the Kocebu Guyot as well as improving “communication” between different water layers. The dominant bacterial taxon across all water layers in the Kocebu Guyot was Gammaproteobacteria, which has been previously associated with marine aggregates of gelatin and detritus, i.e., marine snow (Delong et al., 1993; Schut et al., 1997). Although the sequence dominance does not necessarily mean community dominance because of variations in rDNA copy numbers among species (Zhao et al., 2019; Milivojević et al., 2021), the abundance of Gammaproteobacteria sequences obviously increased with water depth below the DCM layer and peaked in the bottom layer. This kind of vertical pattern generally coincided with the distribution of nitrate (NO_3_-N), phosphate (PO_4_-P), and silicate (SiO_3_-Si), which, together with total and dissolved inorganic carbon, increased with water depth from 200 m to the bottom. These Gammaproteobacteria likely consist of certain obligate chemolithoautotrophs, which show optimal growth under microaerobic conditions in the presence of nitrate in the deep sea (Edwards et al., 2003). This potentially explains the more or less distinct elevation of POC from 1,000 m water depth to the bottom layer in the seamount region (Ma et al., 2020b). According to previous study, the bacteria that attached to large particles showed high taxonomic similarity throughout the water column whereas free-living communities were more isolated vertically (Mestre et al., 2018). These indicate the complex distribution patterns within bacterial communities, which suggests future studies to analyze the particle-attached and free-living bacteria separately for a better understanding of bacterial distribution patterns.

**Figure 7:**
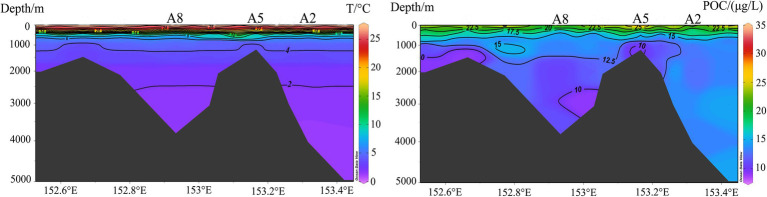
Vertical distribution of water temperature and Particulate organic carbon (POC) in the Kocebu Guyot (modified from Ma et al., 2020b).

In order to assess community connectivity along the vertical gradient, we calculated the proportion of shared OTUs of samples from the same station and same water layer in the Kocebu Guyot and the protist dataset from a previously published article with samples located in non-seamount area of the western Pacific Ocean (Zhao et al., 2020). The higher proportions of shared OTUs from the same water depth in the non-seamount area (mean value 52.8%) compared to those from around the Kocebu Guyot (mean value 30.2%) demonstrate that the seamount introduced disturbances into waters around it and caused exchange of protists between different water layers and thus reduced the shared OTUs in the same water layer. Conversely, samples from the same station in the Kocebu Guyot shared more OTUs compared with those in the non-seamount area, reinforcing that the seamount impacts surrounding waters and enhanced the vertical connectivity of the protist community. Besides, the uplifts of POC concentrations and temperature above summit (Figure 7) also indicate the disturbances introduced by the seamount.

The seamount effect also enhanced vertical interactions within the bacterial community, in that a clear rise of its vertical connectivity was detected around the seamount. The bottom and near-bottom layers of the site located between two seamounts (A8) were particularly significantly connected with the nearby site in layers from 2,000 to 4,000 m. This implies that the complex seamount topography strengthens ocean currents and thus promotes the vertical connectivity of bacterial communities. Likewise, Djurhuus et al. (2017) revealed that the deep layer around seamounts was homogeneous across the entire survey area, and no marked differences were observed between the bacterial communities from samples within depth layers with varying distances from the seabed. The enhanced connectivity along the vertical gradient around seamounts is mainly related to enhanced vertical mixing, internal waves, and mesoscale ocean eddies (White et al., 2007; Read and Pollard, 2017). It is worth noting that this enhanced vertical connectivity could promote the movement of taxa between various water layers, providing ecological opportunities that drive species adaption and diversification (Wellborn and Langerhans, 2015). This is supported by a further study that confirmed a steep increase in the number of observed species within a given sample size in the presence of chaotic advection (Martin et al., 2020).

In our study, we cannot exclude the influence of settling environmental DNA from upper water layers. However, bacterial, protist, and fungal communities were divided into several groups and had significant depth stratifications in our study. The influence of settling environmental DNA did not overwhelm the natural variation among samples. Moreover, the previous studies indicate that DNA appears to be a reliable genetic tool to investigate the diversity and distribution patterns of pelagic microbes (Zhao et al., 2017).

### Factors Shaping Pelagic Distribution and Connectivity Around the Seamount

The bacterial, protist, and fungal communities were characterized by similar vertical stratification patterns, while their community connectivity and the underlying mechanism fluctuated. Different ecological processes had varying degrees of relative importance among pelagic groups. The null model analysis showed that the bacterial, protist, and fungal community of the Kocebu Guyot was largely affected by variable selection and then by homogeneous dispersal, while homogeneous selection took the highest proportion in bacterial community among three taxa. The stronger relative role of homogeneous selection in structuring the bacterial community rather than the protist community is consistent with a previous study that explored mechanisms shaping microbiota in the global upper ocean (Zhao et al., 2019). Nevertheless, unique characteristics were observed in the mechanism structuring seamount microbes around Kocebu Guyot, with drift being less important in shaping both bacterial and protist communities, which is likely due to the presence of the seamount or the sampling depth gradient. The low connectivity within the protist community below 1,000 m and the variable selection dominating the mechanism shaping the protist community in the seamount both indicate that the seamount imposed a hindrance effect on protist dispersal and thus amplified the importance of selection. As opposed to bacterial and protist communities, the fungal community of the Kocebu Guyot was affected by drift to a greater extent, which partly explains the lower connectivity pattern within the fungal community.

With regard to environmental selection, RDA analysis was employed to further determine the environmental factors influencing the pelagic communities. Bacterial, protist, and fungal communities were all significantly structured by depth. Unlike bacteria and protists, fungal community did not exhibit significant correlations with dissolved oxygen. Previous studies revealed that some fungal taxa possessed metabolic adaptations to utilize nitrate and (or) nitrite as an alternative for oxygen (Shoun et al., 1992). Cathrine and Raghukumar (2009) further isolated fungi which were capable of growth under oxygen-deficient conditions, while performing anaerobic denitrification from anoxic marine waters of the Arabian Sea. Planktonic fungi around the seamount may have potential to participate in anaerobic denitrification processes and thus were able to grow in either oxygen-rich or oxygen-deficient water, indicating a weak dependence of the fungal community on the oxygen concentration.

## Conclusion

In the present study, the seamount effect on the diversity, distribution, and connectivity patterns of bacterial, protist, and fungal communities was demonstrated. Bacteria exhibited higher connectivity than protists, whereas an extremely low connectivity was detected for the fungal community, which might be attributed to the parasitic and commensal lifestyles of many fungi. The seamount seemed to not weaken bacterial connectivity, but significantly reduced protist connectivity along the horizontal dimension; its effect also enhanced the vertical connectivity of bacterial and protist communities, aiding the distribution of more taxa to different water layers, and thus providing ecological opportunities to drive the adaptive diversification of species.

Furthermore, the underlying factors shaping the distribution of pelagic microbes around seamount were revealed. The deterministic process influencing bacterial, protist, and fungal community structures had significant correlations with water depth and depth-related factors. Drift appeared to have a more prominent role in shaping the fungal community than the other communities.

## Data Availability Statement

The datasets presented in this study can be found in online repositories. The names of the repository/repositories and accession number(s) can be found at: https://www.ncbi.nlm.nih.gov/bioproject/, PRJNA700132.

## Author Contributions

RZ carried out the samples processing, statistical analyses, original draft writing, and participated in references researching. FZ participated in study designing, statistical analyses, references researching, original draft writing, and manuscript revising. SZ carried out the samples collection. XL provided environmental data. JW provided physical data. KX participated in study designing, manuscript revising, and provided funding support. All authors contributed to the article and approved the submitted version.

## Funding

The research was supported by the National Natural Science Foundation of China (no. 41930533), the Open Fund of CAS Key Laboratory of Experimental Marine Biology, Institute of Oceanology, Chinese Academy of Sciences (no. KF2021NO01), the Strategic Priority Research Program of the Chinese Academy of Sciences (no. XDB42000000) and the Senior User Project of R/V KeXue of the Center for Ocean Mega-Science, Chinese Academy of Sciences (nos. KEXUE2020G08 and KEXUE2019GZ04).

## Conflict of Interest

The authors declare that the research was conducted in the absence of any commercial or financial relationships that could be construed as a potential conflict of interest.

The reviewer JM declared a shared affiliation with the authors to the handling editor at the time of the review.

## Publisher’s Note

All claims expressed in this article are solely those of the authors and do not necessarily represent those of their affiliated organizations, or those of the publisher, the editors and the reviewers. Any product that may be evaluated in this article, or claim that may be made by its manufacturer, is not guaranteed or endorsed by the publisher.
