# Supplementary material for: Bacteria, Protists, and Fungi May Hold Clues of Seamount Impact on Diversity and Connectivity of Deep-Sea Pelagic Communities
Source: Front Microbiol. 2022 Apr 8;13:773487. doi: 10.3389/fmicb.2022.773487 (PMC9024416; doi:10.3389/fmicb.2022.773487)
Supplement: Supplementary file 1 [file Data_Sheet_1.docx]

Supplementary Material


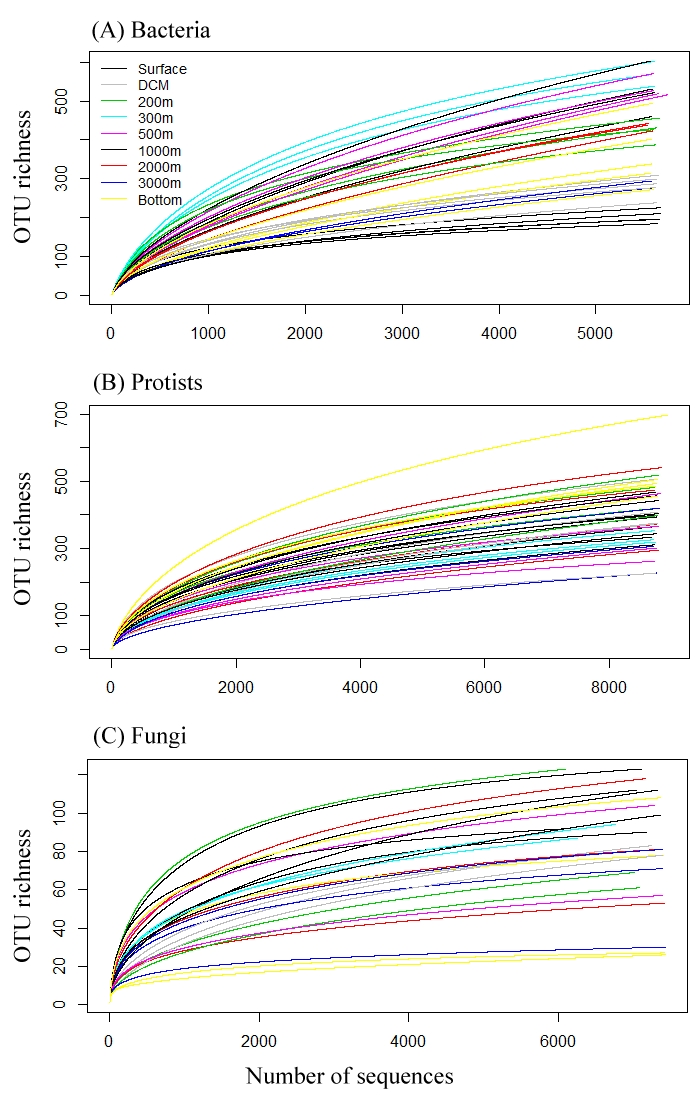


Figure S1. Rarefaction curves of (A) bacteria, (B) protists and (C)fungi.


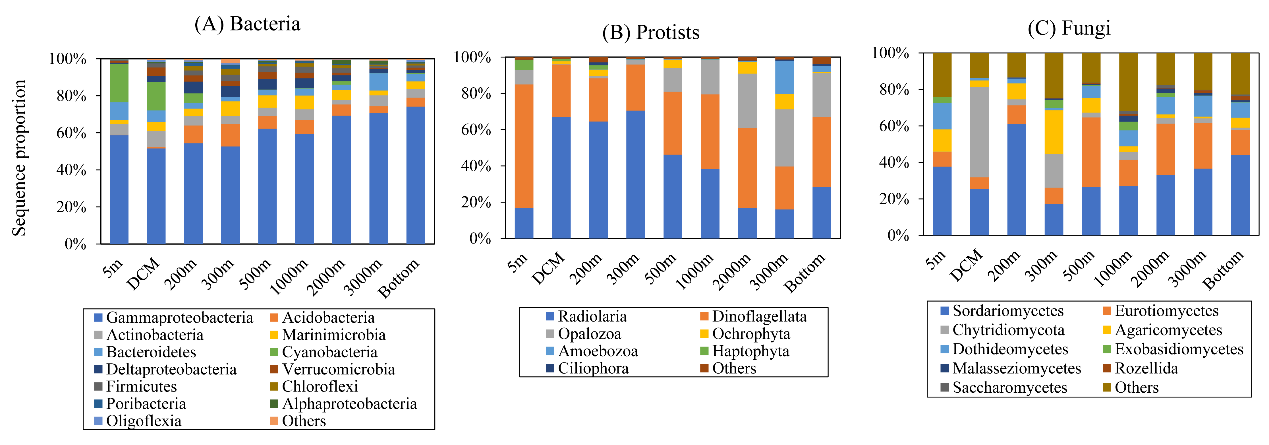


Figure S2. Variations in sequence proportion and their corresponding taxonomic identifications (mainly at the phylum and class level) along the sampling depth range for (A) bacteria, (B) protists, and (C) fungi. The phylum/class with the relative abundance more than 0.5% of bacterial, protists and fungal communities were shown in barplots, others included taxa with the relative abundance less than 0.5%.


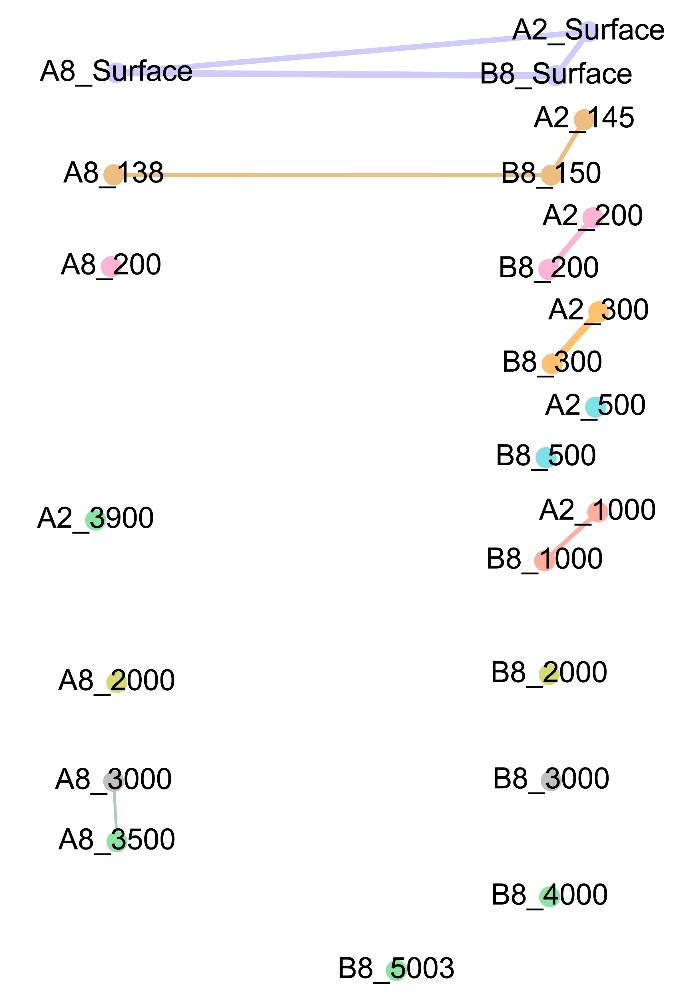


Figure S3. Network depicting correlations of samples in regards of all OTUs of bacteria, protists and fungi. Samples from the same water depth were assigned the same color. Significant correlations (Φ<0.15) are shown by the lines between samples.


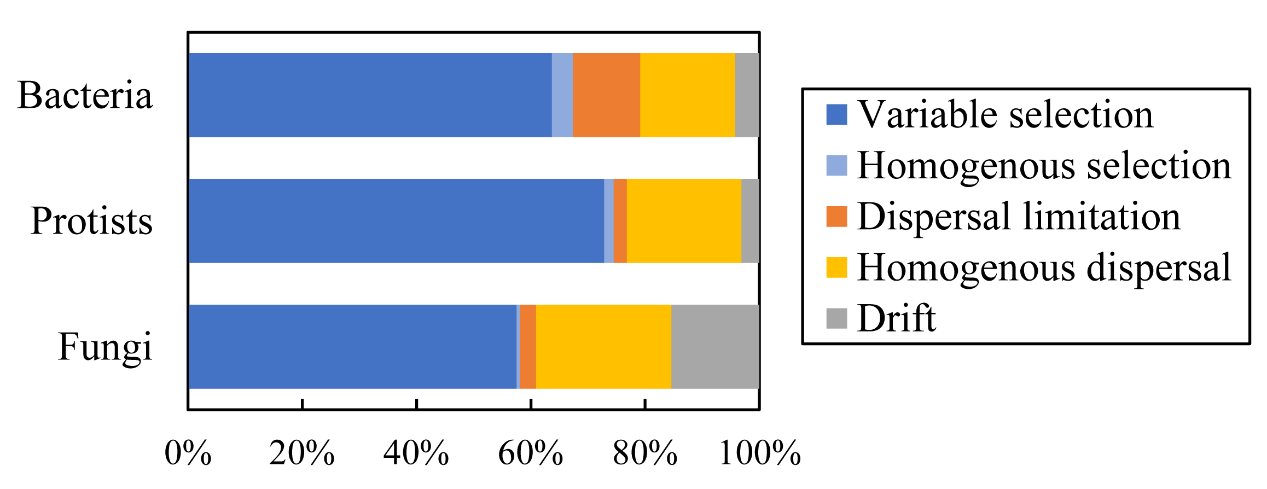


Figure S4. The degree of influence of ecological processes on structuring microbial communities based on the null model analysis.


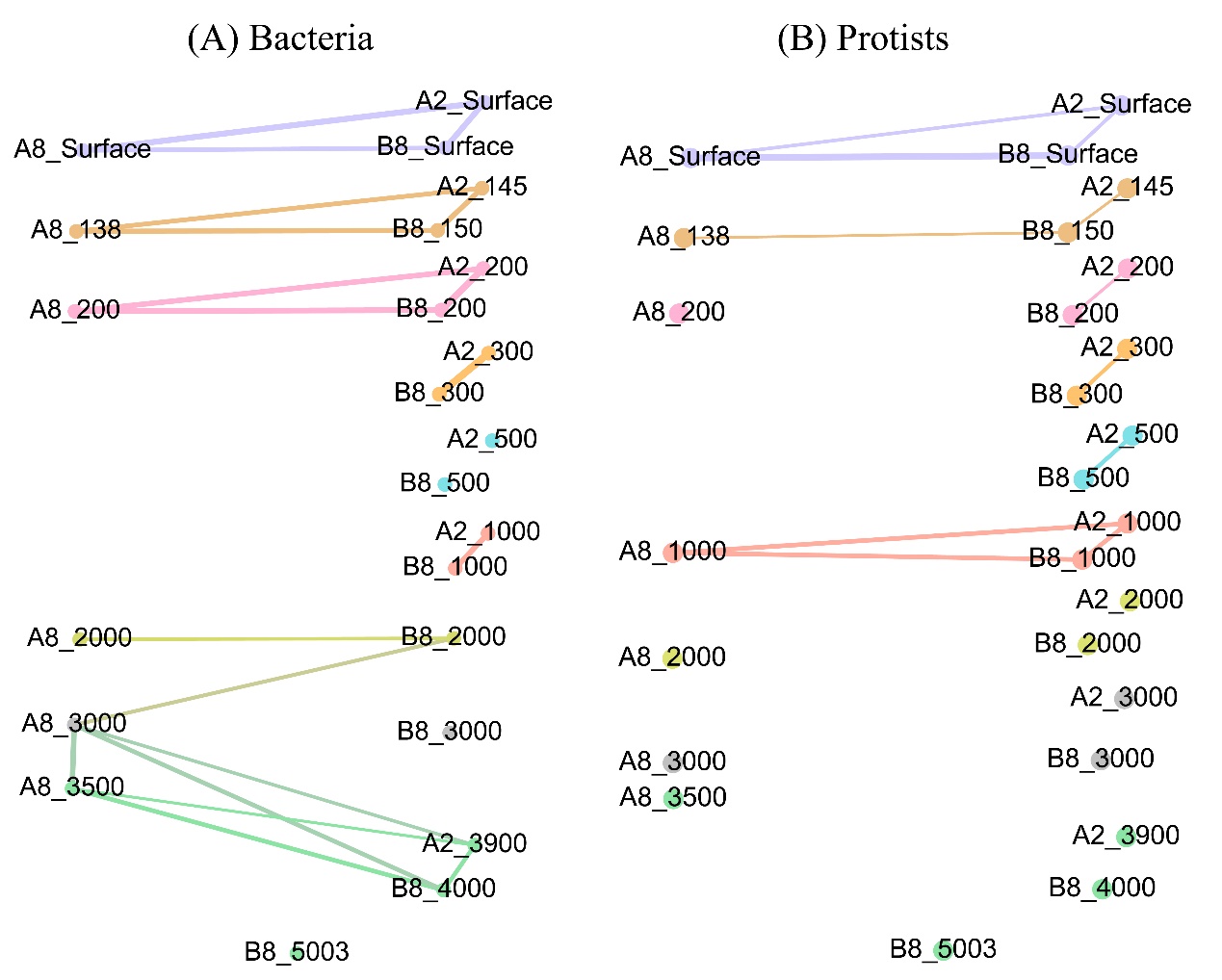


Figure S5. Network depicting correlations of samples in regards of bacteria and protists with the same arrangement of samples as fungi. Samples from the same water depth were assigned the same color. Significant correlations (Φ<0.15) are shown by the lines between samples.


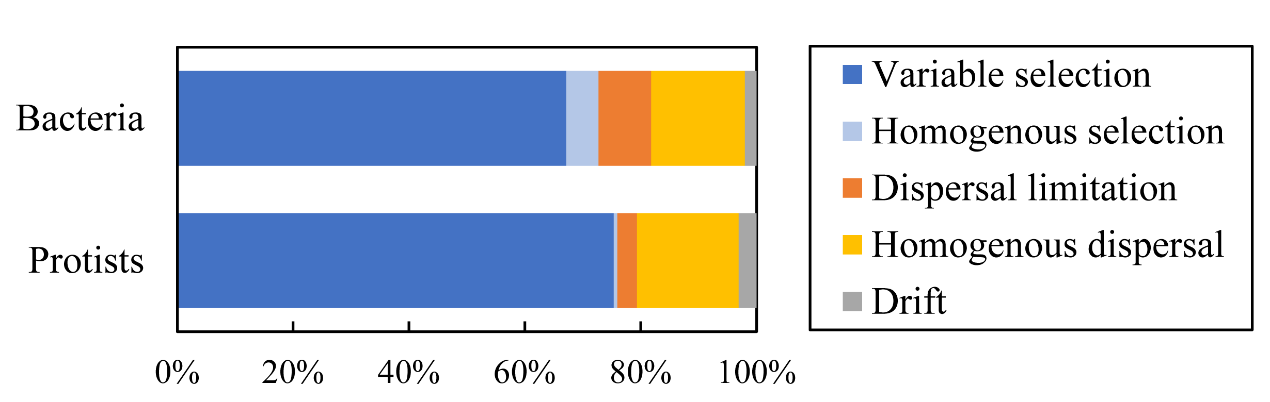


Figure S6. The degree of influence of ecological processes on structuring bacterial and protist communities with the same arrangement of samples as fungal community based on the null model analysis.

Table S1. Names, locations and sampling depths of samples. The samples amplified successfully using 16S, 18S and ITS primers were also listed.

| Sample sites | Samples | Longitude(°E) | Latitude(°N) | Depth(m) | Bacteria  16S | Protists  18S | Fungi  ITS |
| --- | --- | --- | --- | --- | --- | --- | --- |
| A2 | A2_surface | 153.3155 | 17.4030 | 0 | + | + | + |
|  | A2_DCM* |  |  | 145 | + | + | + |
|  | A2_200 |  |  | 200 | + | + | + |
|  | A2_300 |  |  | 300 | + | + | + |
|  | A2_500 |  |  | 500 | + | + | + |
|  | A2_1000 |  |  | 1000 | + | + | + |
|  | A2_2000 |  |  | 2000 |  | + | + |
|  | A2_3000 |  |  | 3000 |  | + | + |
|  | A2_bottom |  |  | 3900 | + | + | + |
| A5 | A5_surface | 153.1643 | 17.3995 | 0 | + | + |  |
|  | A5_DCM |  |  | 165 | + | + |  |
|  | A5_200 |  |  | 200 | + | + |  |
|  | A5_300 |  |  | 300 | + | + |  |
|  | A5_500 |  |  | 500 | + | + |  |
|  | A5_bottom |  |  | 1110 | + | + |  |
| A8 | A8_surface | 152.9332 | 17.3992 | 0 | + | + | + |
|  | A8_DCM |  |  | 138 | + | + | + |
|  | A8_200 |  |  | 200 | + | + | + |
|  | A8_1000 |  |  | 1000 |  | + | + |
|  | A8_2000 |  |  | 2000 | + | + | + |
|  | A8_3000 |  |  | 3000 | + | + | + |
|  | A8_bottom |  |  | 3500 | + | + | + |
| B1 | B1_surface | 153.1689 | 17.7394 | 0 | + | + |  |
|  | B1_DCM |  |  | 105 | + | + |  |
|  | B1_500 |  |  | 500 | + | + |  |
|  | B1_1000 |  |  | 1000 | + | + |  |
|  | B1_2000 |  |  | 2000 | + | + |  |
|  | B1_bottom |  |  | 3200 | + | + |  |
| B8 | B8_surface | 153.1645 | 17.1672 | 0 | + | + | + |
|  | B8_DCM |  |  | 150 | + | + | + |
|  | B8_200 |  |  | 200 | + | + | + |
|  | B8_300 |  |  | 300 | + | + | + |
|  | B8_500 |  |  | 500 | + | + | + |
|  | B8_1000 |  |  | 1000 | + | + | + |
|  | B8_2000 |  |  | 2000 | + | + | + |
|  | B8_3000 |  |  | 3000 | + | + | + |
|  | B8_4000 |  |  | 4000 | + | + | + |
|  | B8_bottom |  |  | 5003 | + | + | + |

*DCM: deep chlorophyll maximum water layer, +: samples amplified successfully

Table S2. Environmental factors shaping bacterial and protist communities with the same arrangement of samples as fungal community, as revealed by RDA analysis. **, *p*<0.01 (significance level of adjusted R^2^)

| Taxa groups | R^2^ (Adjusted) | Significantly affected factors |
| --- | --- | --- |
| Bacteria | 0.576** | PO_4_-P |
|  |  | NO_3_-N |
|  |  | Chl *a* |
|  |  | Depth |
|  |  | Dissolved Oxygen |
| Protists | 0.449** | NO_3_-N |
|  |  | Depth |
|  |  | Chlorophyll *a* |
|  |  | Temperature |
